# Human Mastadenovirus Infections in Children: A Review of the Current Status in the Arab World in the Middle East and North Africa

**DOI:** 10.3390/children9091356

**Published:** 2022-09-06

**Authors:** Fadi S. I. Qashqari

**Affiliations:** Department of Microbiology, College of Medicine, Umm Al-Qura University, Makkah 24381, Saudi Arabia; fsqashqari@uqu.edu.sa; Tel.: +966-53-003-4304

**Keywords:** Arab world, children, human mastadenovirus, infection, Middle East, North Africa

## Abstract

Human mastadenovirus (HAdV) is a non-enveloped icosahedral virus with double-stranded DNA genomes. The mortality rate of HAdV infections can reach 35.5%, while gastroenteritis HAdV infections, HAdV pneumonia, and disseminated disease tend to show a worse outcome, with rates ranging from 44.2% to 50%. In addition, HAdV can cause infections at any age but most commonly in the pediatric population, especially in young children and infants. Therefore, this review aims to assess the current status of HAdV infections among children in the Arab World, particularly in the Middle East and North Africa. Web of Science, Scopus, PubMed, EMBASE, and Google Scholar databases for publications in English were searched up to July 2022 for relevant articles. The literature search yielded a total of 21 studies, which were included in this review. Studies reporting HAdV infections in children were conducted in 17 out of the 22 countries. The average prevalence rate of HAdV infections in children was 12.7%, with average prevalence rates of 12.82% and 12.58% in the Middle East and North African countries, respectively. The highest prevalence rate (28.3%) was reported in Egypt, whereas the lowest prevalence (1.5%) was reported in Sudan. The included studies presented children with signs and symptoms of gastroenteritis, acute respiratory infection, acute diarrhea, and acute hemorrhagic conjunctivitis. In conclusion, the average prevalence rate of HAdV infections in children was 12.7%, with average prevalence rates of 12.82% and 12.58% in the Middle East and North African countries, respectively. Finding the precise prevalence rate of this virus is crucial because it will guide future planning for effective disease control and the selection of particular treatment options during epidemics and special seasons.

## 1. Introduction

Human mastadenovirus (HAdV) is a non-enveloped icosahedral virus with double-stranded DNA genomes [1]. Since Wallace P. Rowe isolated and named the adenoviral serotype HAdV from human tonsils and adenoids in 1953, more than 120 distinct adenoviral serotypes have been discovered in humans, animals, birds, fish, and reptiles. Seven subgroups (A through G) have been assigned to them [2]. HAdV presents a non-enveloped spherical structure [3]. Adenoviral virions often show a crystalline arrangement in infected nuclei [4]. HAdV also contains 26 to 48 kb of linear double-stranded DNA genomes [5]. It creates transcripts of early-stage (E), interim-stage (I), and late-stage (L) regions based on the amount of time that has passed since infection [6]. HAdV is a seasonally transmitted virus that prevails from February to April [7,8,9]. It is a widespread virus that affects humans and is primarily spread by respiratory droplets and feces [10,11,12]. HAdV infections are often seen in military recruits, students, and nursery and kindergarten children, especially children under the age of four, elderly people, and people with compromised immunity [13,14,15].

The clinical manifestations of HAdV infections are very extensive, ranging from a symptom to life-threatening severe respiratory infections and tissue-invasive infections [16,17]. HAdV can cause many symptoms similar to the common cold, including rhinorrhea, fever, cough, and sore throat [18,19]. Bronchitis, bronchiolitis, and pneumonia are examples of lower respiratory diseases that can be serious or even fatal [20]. HAdV infections can also be linked to other conditions such as conjunctivitis, gastroenteritis, cystitis, myocarditis, cardiomyopathy, and meningoencephalitis [21]. A variety of clinical illnesses, including respiratory tract infections, disorders of the respiratory system, keratoconjunctivitis, gastroenteritis, and urinary infections, may be caused by various adenoviral serotypes [22]. Immunocompromised patients are susceptible to invasive adenoviral infection [23]. In immunocompromised patients, HAdV may reactivate or cause new infections, resulting in multi-organ infections like enteritis, hemorrhagic cystitis, hepatitis, and pneumonia [24].

The systematic review and meta-analysis conducted by GU, Jie et al. showed that the mortality rate of AdV infection is 35.5%, while gastroenteritis HAdV infections, HAdV pneumonia, and disseminated disease tend to show a worse outcome, with rates ranging from 44.2% to 50%. However, in the context of immunocompetent patients is associated with a mortality of up to 32.9% [25]. Furthermore, the fatality rates for severe HAdV pneumonia or disseminated disease may exceed 50% in previous reports [25]. In addition, HAdV can cause infections at any age but most commonly in the pediatric population, especially in young children and infants [19]. Invasive HAdV infections do more harm to children [26]. Moreover, by the age of 10 years old, most children have had at least one episode of HAdV infections [19]. Additionally, HAdV testing has produced positive results in 77% of cases of severe acute hepatitis of unknown causes that have been detected in children in many countries [27].

Early diagnosis and supportive therapy are crucial for immunocompromised patients, especially for children [28]. Some large children’s hospitals even recommend HAdV testing as a screening item for children with respiratory tract infections [29,30]. HAdV testing can be performed by isolated cell culture, serological identification, and PCR [31]. However, due to a long time of HAdV cell culture, the limited value of antibody tests in immunosuppressed people, and the lack of quantifying and genotyping functions, the actual clinical application effects are undesirable [32]. By contrast, RT–PCR can be directly used to test nucleotide sequences specific to HAdV pathogens, with high specificity, sensitivity, and desirable genotyping advantages [33]. This also explains why RT–PCR is highly favored. The European Society for Blood and Marrow Transplantation (EBMT) recommends that quantitative PCR (qPCR) be adopted to diagnose and monitor the HAdV in the blood and feces samples of children and adults receiving transplantation so that interventions can be provided as early as possible [34].

Most countries in the Arab World have not yet introduced national HAdV vaccination programs. Although regional and local information on the prevalence and burden of HAdV and strain distribution is important for healthcare practitioners and officials to make appropriate policies and recommendations about HAdV vaccination, there is a remarked lack of comprehensive literature reviews on the prevalence of HAdV infections among children that have been published for the Arab World, particularly in the Middle East and North Africa. Therefore, the purpose of this review is to assess the current status of HAdV infections among children in the Arab World in the Middle East and North Africa. Understanding the current status of HAdV infection prevalence and its associated gastroenteritis, respiratory tract infections, and acute hemorrhagic conjunctivitis is critical to evaluating the performance of surveillance systems where available and to expanding the implementation of these systems.

## 2. Materials and Methods

Electronic literature searches of Web of Science, Scopus, PubMed, EMBASE, and Google Scholar databases for publications in English were systematically conducted without time limit up to July 2022 for studies on HAdV in children in the Arab World in the Middle East and North Africa. A combination of search methods was employed to increase the search scale, namely: (1) search MESH (Human mastadenovirus) using the following terms: “Adenovirus”, “Children”, and “Infection”, and (2) free-text search.

The following were the inclusion requirements: Studies have described the prevalence of HAdV infections in children in the Arab World in the Middle East and North Africa. On the other hand, the following exclusion criteria were considered: (1) reviews; (2) non-English language; (2) case reports; (3) studies not focused on HAdV infections in children; (4) studies that were not performed in the Middle East and North Africa; (4) studies lacking pertinent data; and (4) studies without full text.

The screening procedures were managed, and duplicates were eliminated using EndNote V.X8 software. After removing duplicates, the authors individually the titles, abstracts, and full texts to determine whether the studies were eligible.

The study region, study country, study design, targeted children, sample size, sample location, disease, and HAdV prevalence rate were extracted and recorded using a standardized data collection form that was developed in accordance with the sequence of variables required from the primary source.

## 3. Results

**Figure 1:**
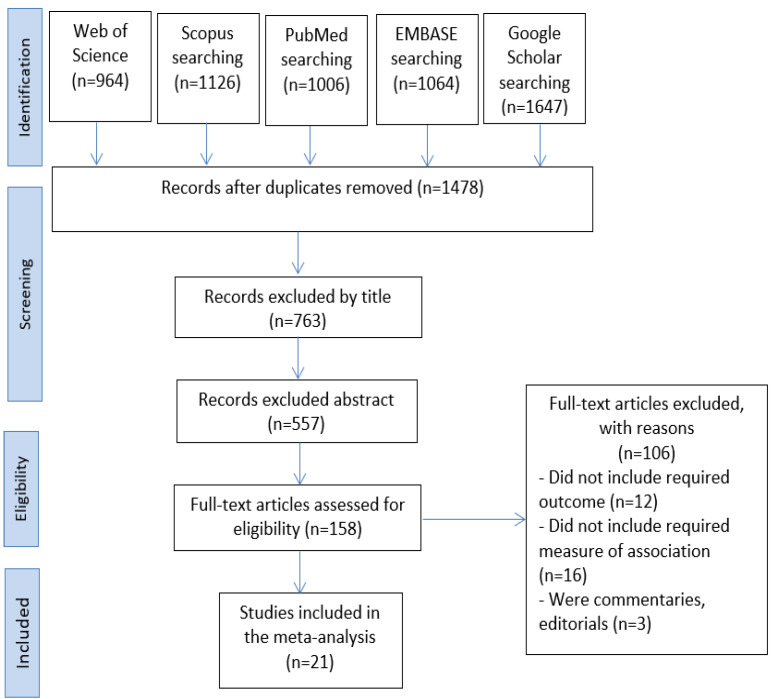
The PRISMA flowchart for the process of selecting and identifying studies.

Our literature search identified 21 articles on HAdV infections in children in the Arab World in the Middle East and North Africa between 2001 and 2021. In total, 5807 articles were found during the search across five databases: Web of Science (*n* = 964), Scopus (*n* = 1126), PubMed (*n* = 1006), EMBASE (*n* = 1064), Google Scholar (*n* = 1647). The duplicates were then removed, leaving 1478 studies. We eliminated 763 studies by title screening and then used abstract screening to eliminate 339 irrelevant studies out of 557. Following this, we read the full texts of the remaining 158 publications and omitted 137 pieces of research since they did not meet our inclusion criteria (Figure 1).

Studies reporting on HAdV infections in children were conducted in 17 out of the 22 countries of the Arab World in the Middle East and North Africa region. We could not find relevant studies published in the following five countries: the Comoros Islands, Djibouti, Mauritania, Somalia, and Syria. Eleven studies were conducted in Middle East countries, namely: Iraq (*n* = 2) [35,36], Jordan (*n* = 2) [37,38], Kuwait (*n* = 2) [39,40], Yemen (*n* = 2) [41,42], Bahrain (*n* = 1) [43], Lebanon (*n* = 1) [44], and the United Arab Emirates (*n* = 1) [45]. Whereas 10 studies were from North African countries, namely: Egypt (*n* = 3) [46,47,48], Morocco (*n* = 2) [49,50], Sudan (*n* = 2) [51,52], Algeria (*n* = 1) [53], Libya (*n* = 1) [54], Tunisia (*n* = 1) [55].

A wide range of HAdV infections in children has been reported in the Arab World in the Middle East and North African countries. The average prevalence rate of HAdV infections in children was 12.7%, with average prevalence rates of 12.82% and 12.58% in the Middle East and North African countries, respectively. The highest prevalence rate of HAdV infections in children (28.3%) was reported in the descriptive cross-sectional observational study conducted in Egypt by Elmahdy Elmahdy et al. 2019, which targeted children suffering from gastroenteritis [47]. On the other hand, the lowest prevalence rate of HAdV infections in children (1.5%) was reported in the descriptive cross-sectional observational study conducted in Sudan by Wafa Elhag et al. 2013, which targeted children under 14 years old with acute diarrhea [51].

Most of the included studies reporting on HAdV infections in children were clinical observational studies. Eleven studies were descriptive cross-sectional observational studies in design; four studies were descriptive prospective cross-sectional studies in design; four studies were descriptive retrospective studies in design; one study was a descriptive case-control observational study, and one study was a case study in design [43].

In all the included studies, HAdV infections in children were reported in hospitalized children. These studies presented children with signs and symptoms associated with gastroenteritis (*n* = 8), acute respiratory infection (*n* = 8), acute diarrhea (*n* = 4), and acute hemorrhagic conjunctivitis (*n* = 1).

A total of 14 studies were carried out among children less than five years of age. The prevalence of HAdV infections among children less than 5 years old was reported in the included studies as follows: Iraq, 14/320 (4.5%) [36]; Iraq, 3/100 (3%) [35]; Jordan, 54/350 (15.4%) [37]; Kuwait, 27/743 (3.6%) [40]; Yemen, 36/326 (11%) [41], United Arab Emirates, 35/203 (17.2%) [45], Egypt, 8/119 (6.7%) [48], Egypt, 20/100 (20%) [46], Egypt, 17/60 (28.3%) [47], Morocco, 119/700 (17%) [49], Sudan, 7/437 (1.6%) [52], Algeria, 9/117 (7.5%) [53], Libya, 17/239 (7.1%) [54], and Tunisia, 3/583 (19.6%) [55]. The remaining studies reported HAdV infections among older children.

There was a noticeable difference in sample sizes between studies and between studies conducted in the same country.

The highest prevalence of HAdV infections among children was in stool samples associated with gastroenteritis for children under 12 years [44] and below ten years [39], at 25.3% and 23.2%, respectively. Whereas the lowest prevalence of HAdV infections among children, 1.5% and 1.6%, was in stool samples associated with acute diarrhea for children below five years in Sudan [51,52] (Table 1).

## 4. Discussion

HAdV infections are one of the most significant etiological agents of serious gastroenteritis, respiratory infections, conjunctivitis, cystitis myocarditis, hemorrhagic cystitis, hepatitis, cardiomyopathy, meningoencephalitis, urinary tract infection, and chronic systemic infections, especially in infants and young children under the age of five [27,39,44,49,53]. This review was conducted to represent the current status of HAdV infections in children in the Arab World in the Middle East and North Africa. Our literature search identified 21 articles on HAdV infections in children in the Arab World in the Middle East and North Africa between 2001 and 2021. Studies reporting on HAdV infections in children were conducted in 17 out of the 22 countries of the Arab World in the Middle East and North Africa region. We could not find relevant studies published in the following five countries: the Comoros Islands, Djibouti, Mauritania, Somalia, and Syria. The main results of this review revealed that the average prevalence rate of HAdV infections in children was 12.7%, with average prevalence rates of 12.82% and 12.58% in the Middle East and North African countries, respectively. The highest prevalence rate of HAdV infections in children (28.3%) was reported in the descriptive cross-sectional observational study conducted in Egypt by Elmahdy Elmahdy et al., 2019, which targeted children suffering from gastroenteritis [47]. On the other hand, the lowest prevalence rate of HAdV infections in children (1.5%) was reported in the descriptive cross-sectional observational study conducted in Sudan by Wafa Elhag et al., 2013, which targeted children under 14 years old with acute diarrhea [51]. In comparison with other countries such as Australia, Brazil, Indonesia, Korea, Iran, the UK, Turkey, Hungary, and Sweden, the prevalence rates of HAdV infections ranged from 1% to 96.3% of patients [53,56,57,58,59,60,61,62,63,64].

Additionally, in this review, a total of 14 studies were carried out among children less than five years of age. The prevalence of HAdV infections among children less than 5 years old was reported in the included studies as follows: Iraq, 14/320 (4.5%) [36]; Iraq, 3/100 (3%) [35]; Jordan, 54/350 (15.4%) [37]; Kuwait, 27/743 (3.6%) [40]; Yemen, 36/326 (11%) [41], United Arab Emirates, 35/203 (17.2%) [45], Egypt, 8/119 (6.7%) [48], Egypt, 20/100 (20%) [46], Egypt, 17/60 (28.3%) [47], Morocco, 119/700 (17%) [49], Sudan, 7/437 (1.6%) [52], Algeria, 9/117 (7.5%) [53], Libya, 17/239 (7.1%) [54], and Tunisia, 3/583 (19.6%) [55]. The remaining studies reported HAdV infections among older children. Differences in prevalence rates in the current review as compared to other studies may be attributed to several reasons, such as the diagnostic technique used for detection, the difference in age of the studied population, and/or the geographical region of the study area. According to the centers for disease control and prevention (CDC), in the United States, the HAdV vaccine is only available for United States military personnel, and there is currently no HAdV vaccine available to the public [65]. Additionally, most countries in the Arab World have not yet introduced national AdV vaccination programs. Therefore, the regional and local information on the prevalence and burden of HAdV and strain distribution is important for healthcare practitioners and officials to make appropriate policies and recommendations about HAdV vaccination.

Moreover, our results showed that in all the included studies, HAdV infections in children were reported in hospitalized children. These studies presented children with signs and symptoms associated with gastroenteritis (*n* = 8), acute respiratory infection (*n* = 8), acute diarrhea (*n* = 4), and acute hemorrhagic conjunctivitis (*n* = 1). Several serotypes of HAdV, but mainly enteric types 40 and 41, have been strongly associated with gastroenteritis in childhood [57]. In addition, HAdV is responsible for three to five percent of all respiratory infections and diarrhea in the USA and a higher percentage in underdeveloped nations [65,66]. These viruses are mostly distributed through the fecal-oral route, and antibodies to them have been found in 50% or more of children under the age of five throughout Asia, Africa, Europe, and South America, with comparable seropositive proportions in the different populations [67,68].

Diarrheal illnesses remain a major public health concern and represent the third leading cause of death globally. In recent years, viral diarrhea has gradually increased in infants and children in both developing and developed countries [69]. Similar to diarrhea brought on by other viruses, HAdV-associated diarrhea may have slightly longer-lasting symptoms. The stools are watery, occasionally red, non-leucocyte-containing, and water-containing [70,71]. Although they are present all year round and everywhere in the world, they are most common in temperate areas in the spring or early summer and once more in the middle of winter [70,71,72]. Additionally, acute hemorrhagic conjunctivitis is a derivative of the highly contagious conjunctivitis virus, otherwise known as pink eye. There are three main viruses that have been studied and confirmed as the agents responsible for acute hemorrhagic conjunctivitis, including enterovirus 70, coxsackievirus A24 variant (CA24v), and HAdV [73].

Finally, this review demonstrated that the highest prevalence of HAdV infections among children was in stool samples associated with gastroenteritis for children under 12 years [44] and below ten years [39], at 25.3% and 23.2%, respectively. Whereas the lowest prevalence of HAdV infections among children, 1.5% and 1.6%, was in stool samples associated with acute diarrhea for children below five years in Sudan [51,52]. It appears that HAdV infection is a significant hygiene problem in these nations. Finding the precise prevalence rate of this virus is crucial since it will aid in future programming for the right control and selection of specialized treatment approaches for the illness during unique seasons and the HAdV outbreak. Furthermore, to limit the spread of infection, it is important to maintain infectious control practices and educate the infected individual to avoid sharing towels, glasses, or any other item in contact with the eyes. Additionally, these individuals should stay at home and avoid going to work or school when symptomatic in order to prevent spreading the infection.

The main strength of the current review is being the first review that shows the current status of HAdV infections among children in the Arab World in the Middle East and North Africa. However, our study has a number of limitations. The research population, times, locations, data collection techniques, and clinical disease care could all contribute to differences in estimates between and within countries. Because there were few studies conducted in each of these nations, the findings indicating the prevalence of HAdV infections may not be generalizable to several Arab nations. Some studies were carried out at large hospitals in urban areas, so the results might not be applicable to rural or other settings.

Additionally, the surveillance techniques used in the numerous published publications differ from country to country, and variances in the estimations may result from variations in HAdV detection techniques. Nevertheless, the overall results typically support earlier literature; thus, any systematic error is improbable. Finally, our study did not study the seasonality of HAdV prevalence.

## 5. Conclusions

In conclusion, the average prevalence rate of HAdV infections in children was 12.7%, with average prevalence rates of 12.82% and 12.58% in the Middle East and North African countries, respectively. Finding the precise prevalence rate of this virus is crucial because it will guide future planning for effective disease control and the selection of particular treatment options during epidemics and special seasons.

## Figures and Tables

**Table 1:** Reported HAdV infections among children in the Arab World, particularly in the Middle East and North Africa, up to July 2022.

Region	Country	Study	Study Design	Targeted Children	Sample Size	Sample Location	Disease	**Prevalence Rate**
Middle East	Iraq	Ali Harb et al., 2019	A descriptive cross-sectional observational study	Children below five years with acute diarrhea	320 children	Stool specimens	Gastroenteritis	4.5%
	Iraq	Dilshad Jaff et al., 2016	A descriptive cross-sectional observational study	Children below five years of age with gastroenteritis	100 children	Stool specimens	Gastroenteritis	3.0%
	Jordan	Nasser Kaplan et al., 2008	A descriptive prospective cross-sectional study	Children below five years with an acute respiratory infection	326 children	Nasopharyngeal aspirates	Acute respiratory infection	18.0%
	Jordan	Mamdoh Meqdam et al., 2001	A descriptive cross-sectional observational study	Children below thirteen years old with respiratory tract infections	350 children	Nasopharyngeal aspirates	Acute respiratory infection	15.4%
	Kuwait	Hawraa Mohammad et al., 2020	A 4-year descriptive retrospective study	Children below ten years with gastroenteritis	84 children	Stool samples	Gastroenteritis	23.2%
	Kuwait	Wassim Chehadeh et al., 2018	A 4-year descriptive retrospective study	Children below four years with severe respiratory disease	743 children	Nasopharyngeal aspirate and Nasopharyngeal swab	Severe respiratory infections	3.6%
	Yemen	Khaled Al-Moyed et al., 2015	A descriptive cross-sectional observational study	Children below five years with acute gastroenteritis	326 children	Stool samples	Gastroenteritis	11%
	Yemen	Mohammad Al Amad et al., 2019	A descriptive retrospective study	Children below fifteen years of age with severe acute respiratory infections	1413 children	Nasopharyngeal and oropharyngeal swabs	Acute respiratory infection	7.0%
	Bahrain	Aysha Agab et al., 2016	A case study	A seven-year-old male and a five-year-old female	Two Bahraini siblings	Conjunctival swabs	Acute hemorrhagic conjunctivitis	NM
	Lebanon	Rasha Zaraket et al., 2020	A 12-months descriptive retrospective study	Children below twelve years with acute gastroenteritis	308 children	Stool samples	Gastroenteritis	25.3%
	United Arab Emirates	Ahmed Alsuwaidi et al., 2021	A descriptive case-control observational study	Children below five years with diarrhea	203 children as a case	Stool samples	Acute diarrhea	17.2%
North Africa	Egypt	Abdou Kamal Allayeh et al., 2018	A descriptive cross-sectional observational study	Children below five years with gastroenteritis	119 children	Fecal diarrhea samples	Gastroenteritis	6.7%
	Egypt	Maysaa El Sayed Zaki et al., 2017	A descriptive cross-sectional observational study	Children below five years with acute diarrhea	100 children	Stool sample	Acute diarrhea	20.0%
	Egypt	Elmahdy Elmahdy et al., 2019	A descriptive cross-sectional observational study	Children below five years with gastroenteritis	60 children	Stool samples	Gastroenteritis	28.3%
	Morocco	Imane Jroundi et al., 2014	A descriptive prospective cross-sectional study	Children below five years with respiratory symptomatology	700 children	Nasopharyngeal aspirates	Acute respiratory infection	17.0%
	Morocco	Marcil Sarrah et al., 2018	A descriptive prospective cross-sectional study	Children below fourteen years with severe acute viral respiratory infections	103 children	Nasopharyngeal aspirates	Acute respiratory infection	16.5%
	Sudan	Wafa Elhag et al., 2013	A descriptive cross-sectional observational study	Children below fourteen years old with acute diarrhea	511 children	Stool samples	Acute diarrhea	1.5%
	Sudan	Mosab Adam et al., 2018	A descriptive cross-sectional observational study	Children below five years with acute diarrhea	437 children	Stool samples	Acute diarrhea	1.6%
	Algeria	Fawzi Derrar et al., 2019	A descriptive prospective cross-sectional study	Children below two years with respiratory tract infections	117 children	Nasal or nasopharyngeal aspiration	Acute respiratory infection	7.5%
	Libya	Amal Rahouma et al., 2011	A descriptive cross-sectional observational study	Children below five years with acute diarrhea	239 children	Stool specimens	Gastroenteritis	7.1%
	Tunisia	Ines Brini et al., 2020	A descriptive cross-sectional observational study	Children below five years with acute respiratory infections	583 children	Nasopharyngeal aspirate	Acute respiratory infection	19.6%

NM: denote to not mentioned.

## Data Availability

Not applicable.
